# Multi-Channel Gating Chip in 0.18 µm High-Voltage CMOS for Quantum Applications

**DOI:** 10.3390/s23249644

**Published:** 2023-12-06

**Authors:** Christoph Ribisch, Michael Hofbauer, Seyed Saman Kohneh Poushi, Alexander Zimmer, Kerstin Schneider-Hornstein, Bernhard Goll, Horst Zimmermann

**Affiliations:** 1 Institute of Electrodynamics, Microwave and Circuit Engineering, Faculty of Electrical Engineering and Information Technology, Technische Universität Wien, 1040 Vienna, Austria; michael.hofbauer@tuwien.ac.at (M.H.); saman.kohneh@tuwien.ac.at (S.S.K.P.); kerstin.schneider-hornstein@tuwien.ac.at (K.S.-H.); bernhard.goll@tuwien.ac.at (B.G.); horst.zimmermann@tuwien.ac.at (H.Z.); 2 X-FAB, 99097 Erfurt, Germany; alexander.zimmer@xfab.com

**Keywords:** single-photon avalanche diode, SPAD, gating circuit, CMOS, quantum simulator

## Abstract

A gating circuit for a photonic quantum simulator is introduced. The gating circuit uses a large excess bias voltage of up to 9.9 V and an integrated single-photon avalanche diode (SPAD). Nine channels are monolithically implemented in an application-specific integrated circuit (ASIC) including nine SPADs using 0.18 µm high-voltage CMOS technology. The gating circuit achieves rise and fall times of 480 ps and 280 ps, respectively, and a minimum full-width-at-half-maximum pulse width of 1.26 ns. Thanks to a fast and sensitive comparator, a detection threshold for avalanche events of less than 100 mV is possible. The power consumption of all nine channels is about 250 mW in total. This gating chip is used to characterize the integrated SPADs. A photon detection probability of around 50% at 9.9 V excess bias and for a wavelength of 635 nm is found.

## 1. Introduction

Avalanche photodiodes operated above their breakdown voltage (in the Geiger mode) are able to detect single photons and are therefore called single-photon avalanche diodes (SPADs). The SPAD is biased with a reverse voltage of V_R_ = V_BD_ + V_EX_, where V_BD_ is the breakdown voltage and V_EX_ is the so-called excess bias voltage. If a photon hits the SPAD, a detectable current is generated. This is possible because the electric field in the multiplication layer is so high that a single electron–hole pair can trigger a self-sustaining avalanche [1,2]. This avalanche needs to be quenched, i.e., the voltage across the SPAD has to be reduced to or below the breakdown voltage, to enable the SPAD to detect the next photon. The quenching is also important because the reverse current through the SPAD could rise to high values and damage the SPAD. After the dead time, the SPAD is again recharged to V_BD_ + V_Ex_ and is ready to detect the next photon.

There are different methods of quenching SPADs, such as passive, active, mixed and gated [3]. Passive quenching is very simple because a high-value resistor in series to the SPAD reduces the reverse voltage of the SPAD if the current rises. After the quenching, the reverse voltage slowly increases again. Active quenching circuits almost immediately quench the SPAD after an avalanche is detected. Fast active quenching reduces the after-pulsing probability [4]. Mixed quenching circuits use the passive and the active part. In the gated mode, the SPAD is charged and quenched with a defined rate.

The gated mode is ideal if the arrival time of the photons is known and periodic [5,6,7]. The gating mode reduces possible dark counts and also reduces the after-pulsing probability (which would have been caused by prior photon absorptions or dark counts) compared to using an active quenching circuit) [8,9]. This mode is therefore best suitable for the detection of the outcoming photons of a quantum simulator [10].

Also important for the application of SPADs in a quantum simulator is a high photon detection probability (PDP) to increase the rate of valid calculations, since this rate depends on PDP to the power of the number of Qubits, i.e., number of photons (Supplementary Information in [11]). The PDP is known to increase with the excess bias voltage V_EX_ [8,12]. However, DCR and APP also increase with V_EX_. The APP is proportional to the capacitance of the SPAD (more precisely to the total capacitance of the input node of the quencher or gater including the SPAD capacitance) [4]. With SPADs integrated on the quencher or gater chip, the input-node capacitance can be reduced by elimination of the bond pad capacitances, which would be present in the case of off-chip SPADs. Thus, there is a need for fast switching transistors, which favor a small-structure-size technology, and for high breakdown voltages. Both requirements necessitate a compromise and/or a sophisticated circuit topology like cascoding [13]. In recent years, we implemented cascoded switching transistors in active quenchers [14,15] and gaters [16], both in 0.35 µm CMOS technology, to increase the excess bias voltage far above the nominal supply voltage. In this work, we use 0.18 µm high-voltage CMOS and double cascoding for the realization of a gating chip with nine channels, each with an integrated SPAD, to obtain shorter rise and fall times. To the best of the authors’ knowledge, the proposed gating circuit is the first to use double cascoding and supply a maximum excess bias voltage of 9.9 V.

Section 2 describes the gater chip, the SPAD is introduced in Section 3, the gating circuit is explained in Section 4, the SPAD is characterized in Section 5 and a comparison and conclusions are drawn in Section 6.

## 2. Gater Chip

**Figure 1:**
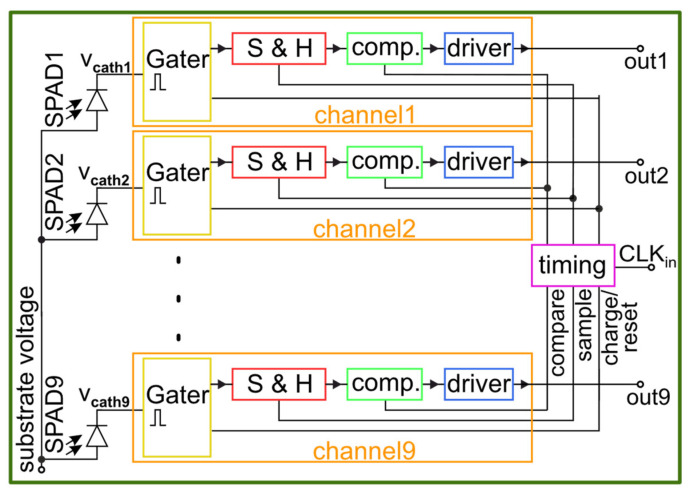
Block diagram of gating chip with 9 channels and integrated SPADs.

Figure 1 shows the simplified block diagram of the multi-channel gater chip. The proposed system consists of a timing section (violet) and nine separate channels (orange), which all include a double-cascoded gater switch (yellow), a sample and hold stage (red), a sensitive comparator (green) and a series of digital output drivers (blue). The circuit is designed with 1.8 V and 3.3 V MOSFETs in high-voltage 180 nm CMOS technology. The 3.3 V transistors are used to reach a voltage swing of 9.9 V at the cathodes of the SPADs and the faster 1.8 V transistors are employed for all other parts to ensure the best possible timing performance and to reduce power consumption. The driver section of every channel is split into multiple smaller drivers to send the signal across the hole chip with a total metal track length of about 5 mm for each channel. Directly next to the corresponding digital output bonding pad, an output driver is placed.

The timing signals for every channel are generated in a central timing block (violet) and distributed via matched delay lines to the channels, which are up to 1 mm away from the timing block. Every channel has a separate bias voltage input for the comparator reference to cope with process and temperature variations. To adjust the performance of the sample and hold stages, a separate bias voltage is used, which is shared to all channels to reduce the number of necessary bond wires. The charging duration of the SPADs and the gating window duration can be adjusted via two separate bias voltages. These voltages control variable delays in the timing section and therefore affect all channels simultaneously. It is possible to charge the SPADs to a lower voltage (V_EX_ < 9.9 V) and therefore also to reach shorter gating windows.

## 3. Structure of SPAD

**Figure 2:**
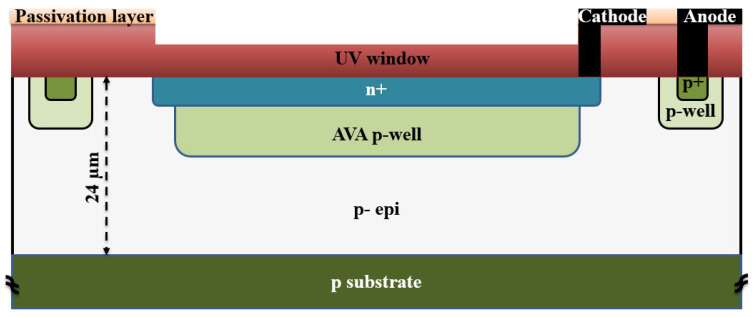
Schematic cross section of the n+/avalanche-p-well CMOS SPAD (not to scale).

Figure 2 shows the cross-section of the integrated SPAD fabricated in a 0.18 μm high-voltage CMOS process. The SPAD comprises a shallow n+ region and a customized p-well avalanche region formed on a p-type doped epitaxial layer (p- epi) with a doping concentration of ~1.3 × 10^13^ cm^−3^ and a thickness of ~24 μm. The thick epi layer is advantageous for a low capacitance (i.e., low APP) and for a high PDP for red and near-infrared light. The diameter of the p-well (50 µm) is considered smaller than that of the n+ cathode (55 µm) to avoid early edge breakdown. An STI with a width of 10.1 µm separates the n+ cathode and the p+ anode ring.

**Figure 3:**
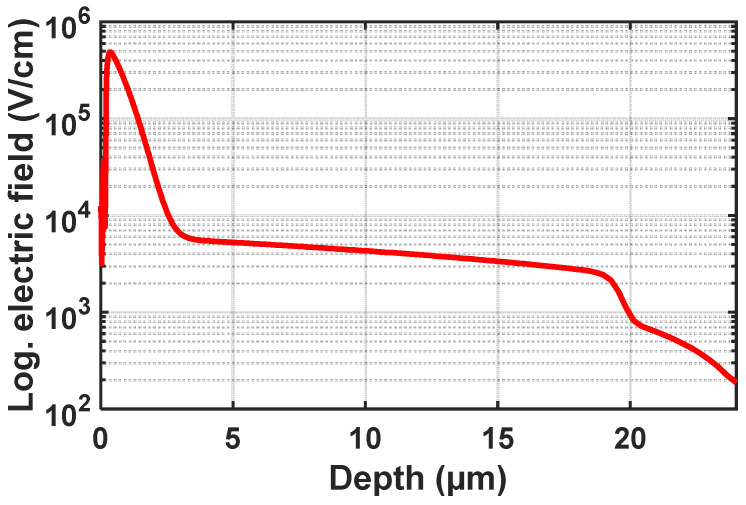
A vertical cross-section of the electric field at the center of the structure at an excess bias of 10 V.

This design features a thick absorption region. When the device is reverse-biased beyond the breakdown voltage, a high electric field is formed at the n+/p-well junction that serves as an avalanche multiplication zone. At this voltage level, a weaker electric field expands from below the p/n junction through the p-well and through the p- epitaxial layer towards the substrate, which serves as the absorption region. All electrons photogenerated in the thick absorption region drift up to the p-well and will pass through the full thickness of the multiplication zone for impact ionization, resulting in a high PDP. The breakdown voltage of this device is 34.4 V according to simulations with Silvaco Atlas [17]. The simulated capacitance of the SPAD is 20 fF. Figure 3 shows a vertical cross-section of the electric field at an excess bias voltage of 10 V, as obtained through Geiger mode device simulation using Atlas.

The electric field strength is larger than 3000 V/cm throughout the thick absorption zone/epitaxial layer. This results in an electron drift velocity larger than 4 × 10^6^ cm/s, leading to a carrier collection time of less than 0.5 ns. Thus, a very short gating pulse at the nanosecond scale should be possible.

## 4. Gating Circuit

### 4.1. High-Voltage Cascode Switch

**Figure 4:**
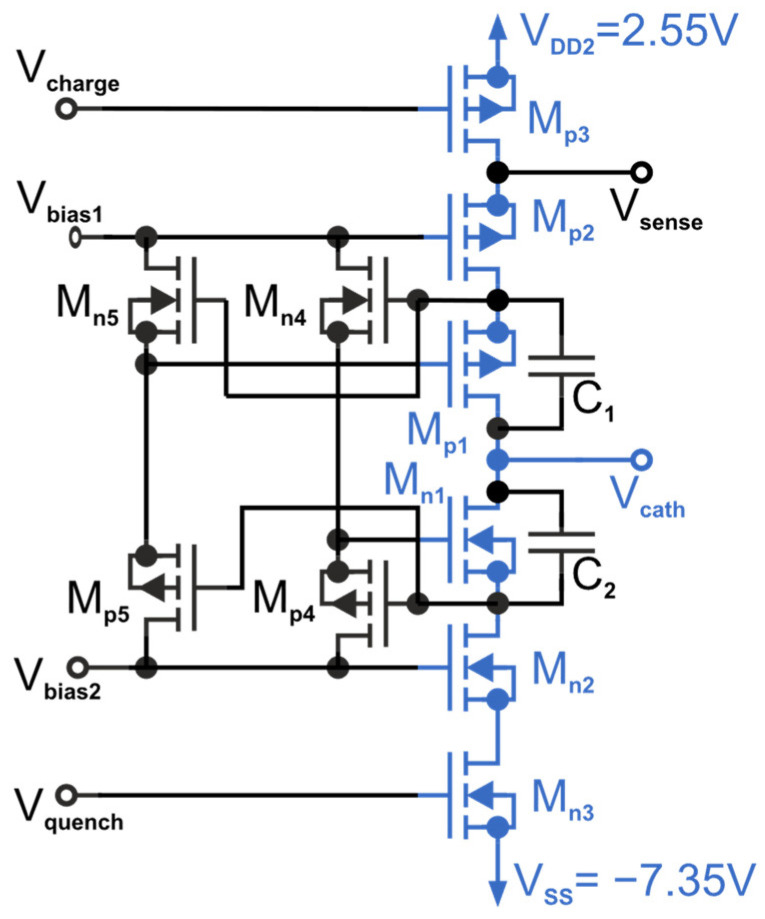
High-voltage double-cascoded switch (HVCS) with dual adaptive bias shift.

The proposed gating switch for a maximum excess bias of 9.9 V is shown in Figure 4 (marked blue; a modified version of the quenching switch of an active quencher realized in 0.35 µm CMOS and presented in [15]). It is a high-voltage double-cascoded switch (HVCS) with dual adaptive bias voltages. The simple adaptive gate bias of the second cascode transistor used in [15] had to be extended to the dual adaptive gate biasing with a separate adaptive bias with M_n4_ and M_p4_ for M_n1_, as well as M_n5_ and M_p5_ for M_p1_, to keep the voltages during the transition phases in their specified voltage limits. The HVCS is built with standard 3.3 V transistors. The bulks of the MOSFETs M_p1_–M_p3_ and M_n1_–M_n2_ are connected to their corresponding sources. The three stacked PMOS MOSFETs M_p1_–M_p3_ have to withstand 9.9 V, when the SPAD is quenched and the transistors M_n1_–M_n3_ pull the cathode to −7.35 V. The three stacked NMOS MOSFETs M_n1_–M_n3_ also have to sustain 9.9 V when the SPAD is charged to 9.9 V and waiting for a photon to arrive. Three transistors for each side are required since the nominal voltage of a single transistor is 3.3 V. The cathode of the SPAD is connected to V_cath_ and can therefore be switched between −7.35 V and 2.55 V (9.9 V swing). All transistors are isolated to substrate with deep n-wells. To save space, the MOSFETs M_n1_–M_n3_ as well as M_n5_ and M_n4_ are in the same deep n-wells. Due to the high voltage difference between the wells and the substrate all corners of the deep n-wells are rounded. The corners of the p-wells of the transistors M_n1_, M_n2_, M_n3_, M_n4_, and M_n5_ are also rounded to withstand the occurring higher voltages than the nominal 8 V between the p-wells and the deep n-wells.

If quenching is initiated (V_quench_ = V_bias2_) and charging is deactivated (V_charge_ = V_DD2_), the transistor M_n3_ switches on and the transistor M_p3_ switches off. The drains of M_n3_ and M_p3_ are discharged to −7.35 V and V_bias1_ + V_Th_, respectively. The transistors M_n2_ and M_p2_, respectively, switch on and off because the gate of M_p2_ is at a fixed bias voltage of V_bias1_ = −0.75 V and the gate of M_n2_ is at a fixed bias voltage of −4.05 V. The drain of M_n2_ is discharged to V_ss_ = −7.35 V. Therefore, the transistors M_p4_ and M_p5_ are in the on-state and shift the voltages on the gate of M_n1_ and M_p1_ to V_bias2_ = −4.05 V. This shift at the gate of M_n1_ sets this transistor into the on-state and discharges its drain, and therefore the V_cath_ output to −7.35 V. Because of the shift at the gate of M_p1_ (caused by M_n4_ and M_p4_), the transistor M_p1_ is now in the off-state. The adaptive bias circuitry (M_n4_, M_n5_, M_p4_, M_p5_) is necessary to keep the voltages on M_n1_ and M_p2_ in the process limits. To keep the potential difference on the transistors M_p1_ and M_n1_ during the fast-switching process also in the range of the absolute maximum voltages, two slightly different adaptive bias voltages with slightly different time behavior are used (dual adaptive bias; M_p5_–M_n5_ and M_p4_–Mn_p4_). The capacitors C_1_ and C_2_ act in combination with the trace resistance as a snubber in [18] to suppress voltage peaks at the blue-marked transistors occurring during the transition between V_DD2_ and V_ss_. Without them, voltage peaks over 5 V would occur.

### 4.2. Sample and Hold and Comparator

**Figure 5:**
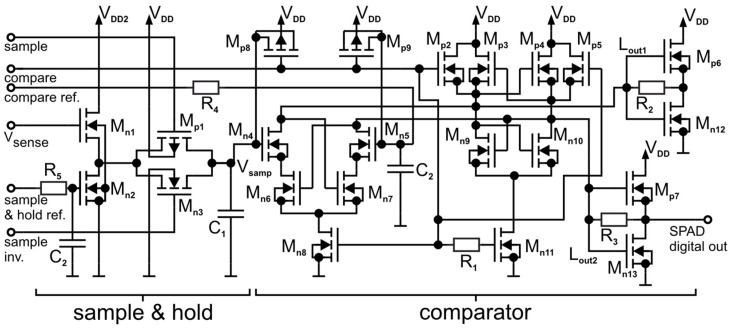
Comparator-, sample- and hold stage-inclusive level shifting (M_n1_ is a 3.3 V MOSFET; all other transistors are 1.8 V MOSFET).

The next stage after the high-voltage cascode switch is the sample and hold circuitry, which is followed by the comparator stage as shown in Figure 5. The voltage on the SPADs cathode is indirectly sensed at the drain of M_p3_ at the HVCS (Figure 4). The voltage range of V_sense_ is between V_bias1_ = −0.75 V and V_DD2_ = 2.55 V, which is a 3.3 V range. To obtain the best possible timing performance and to save energy in the following stages, 1.8 V transistors are used, and only M_n1_ in Figure 5 is therefore a 3.3 V MOSFET. A source follower with a current source load (M_n1_ and M_n2_) is used to shift the voltage range down into the range of 0 V–1.8 V. The “sample & hold ref.” bias input is used to control the current through the source follower and to shift the voltage externally to manage process, voltage, and temperature variations. All nine channels get the same reference voltage to reduce the needed number of pads. The resistor R_5_ and the capacitor C_2_ act as a low pass filter to reduce the noise and distortion coming from very long metal traces (up to 7 mm).

The hold capacitance C_1_ is charged to approximately V_sense_ when the gating window is open, and the transmission gate M_n3_/M_p1_ conducts. The transmission gate (M_p1_ and M_n3_) is used to disconnect the capacitor C_1_ just before the SPAD is quenched to store the value of V_sense_. The stored voltage V_samp_ is an indicator if an avalanche was triggered because it indicates if V_sense_ and therefore V_cath_ were discharged due to an avalanche. The control signals “sample” and “sample inv.” are generated centralized for all channels in the timing section (see Figure 1). The signals are cross-coupled to ensure a simultaneous transition on both transistors of the transmission gate.

The proposed clocked high-speed comparator (modified compared to that realized in 65 nm CMOS and presented in [16]) is also shown in Figure 5 on the right side. It consists of a latch (M_p3_, M_n9_, M_p4_, M_n10_) with two reset MOSFETs M_p2_ and M_p5_, the input transistors M_n4_ and M_n5_, two cross-coupled MOSFETS M_n6_ and M_n7_, two tail transistors M_n8_ and M_n11_ and two output CMOS inverters with a feedback resistor (M_p6_, M_n12_, M_p7_, M_n13_). Only the output of one inverter (M_p7_, M_n13_) is used. The other is used to ensure a symmetrical load at the latch nodes (L_out1_, L_out2_). The resistive feedback at the CMOS inverters reduces the slope of the transfer characteristics and therefore reduces the risk of switching the next logic stage on and off multiple times during the decision phase.

In the reset phase (compare = 0 V), M_n8_ and M_n11_ are switched off and the transistors M_p2_ and M_p5_ reset the output nodes of the latch to V_DD_. In the compare phase (compare = V_DD_ = 1.8 V), the reset transistors M_p2_ and M_p5_ are switched off and M_n8_ and M_n11_ are switched on to enable the input transistors to decide in which state the latch is pulled. M_n6_ and M_n7_ are switched on from the rest phase because their gates are pulled to V_DD_. M_n6_ and M_n7_ reduce the energy consumption by avoiding static current. In addition, the low-power process module was used. The input MOSFETs M_n4_ and M_n5_ discharge the nodes L_out1_ and L_out2_. The voltages at gates of M_n4_ and M_n5_ determine if L_out1_ or L_out2_ is discharged fast and determine therefore if the sampled voltage (V_samp_) or the reference voltage (compare ref.) is lower. When the reference voltage is higher than the sampled voltage V_samp_, the node L_ou2_ is discharged faster via M_n10_ and M_n5._ If the node L_out2_ is equal to V_DD_–V_th_ (V_th_ of M_p3_ and M_p4_), M_p3_ enters into the on mode and the complete latch switches because of the positive feedback to L_ou1_. The reference voltage is filtered and stabilized with the resistor R_4_ and the capacitor C_2_ which act as a low pass filter to reduce the noise and distortion coming from the very long metal traces. To compensate charge injection during the change from reset to the compare phase and therefore obtain a more sensitive comparator, M_p8_ and M_p9_ (MOSCAPs) inject charge during the transition for compensation purposes.

The clock for the comparator (compare) is centralized and generated for all channels in the timing section (see Figure 1). The compare phase is triggered approximately 1 ns after the transmission gate is closed to reduce distortions from the quenching process to achieve a higher sensitivity. The reset phase is triggered by the negative edge of the chip’s clock input to avoid distortions in the charging phase caused by the big digital output drivers on the chip. The resistor R_1_ causes a short delay between the gate of M_n11_ and the other tail transistor M_n8_ and the reset transistors (M_p2_ and M_p5_). This delay of the compare signal (clock) is caused by the resistance of R_1_ and the M_n11_ gate capacitance. This short delay retards the discharging of L_out1_ and L_out2_ and therefore increases ΔU_0_, which increases the robustness of the latch against noise and mismatch. ΔU_0_ is the initial voltage difference at the latch nodes L_out1_ and L_out2_ which causes the latch to regenerate [19].

### 4.3. Layout

**Figure 6:**
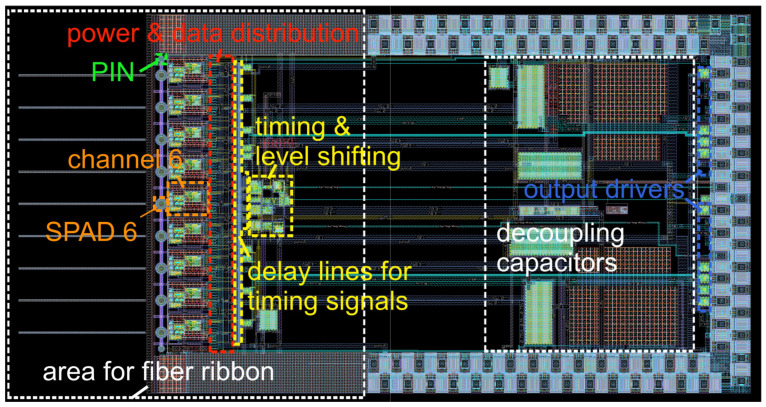
Layout of the gater chip containing 9 integrated 50 µm SPADs.

**Figure 7:**
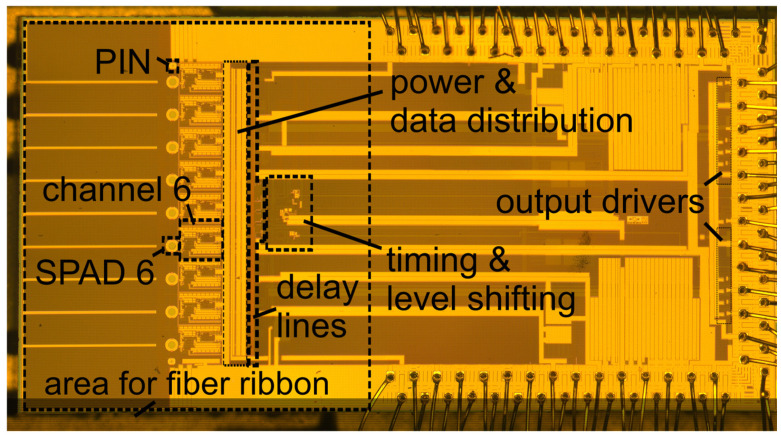
Photo of the complete multi-channel gating chip with bonding wires.

**Figure 8:**
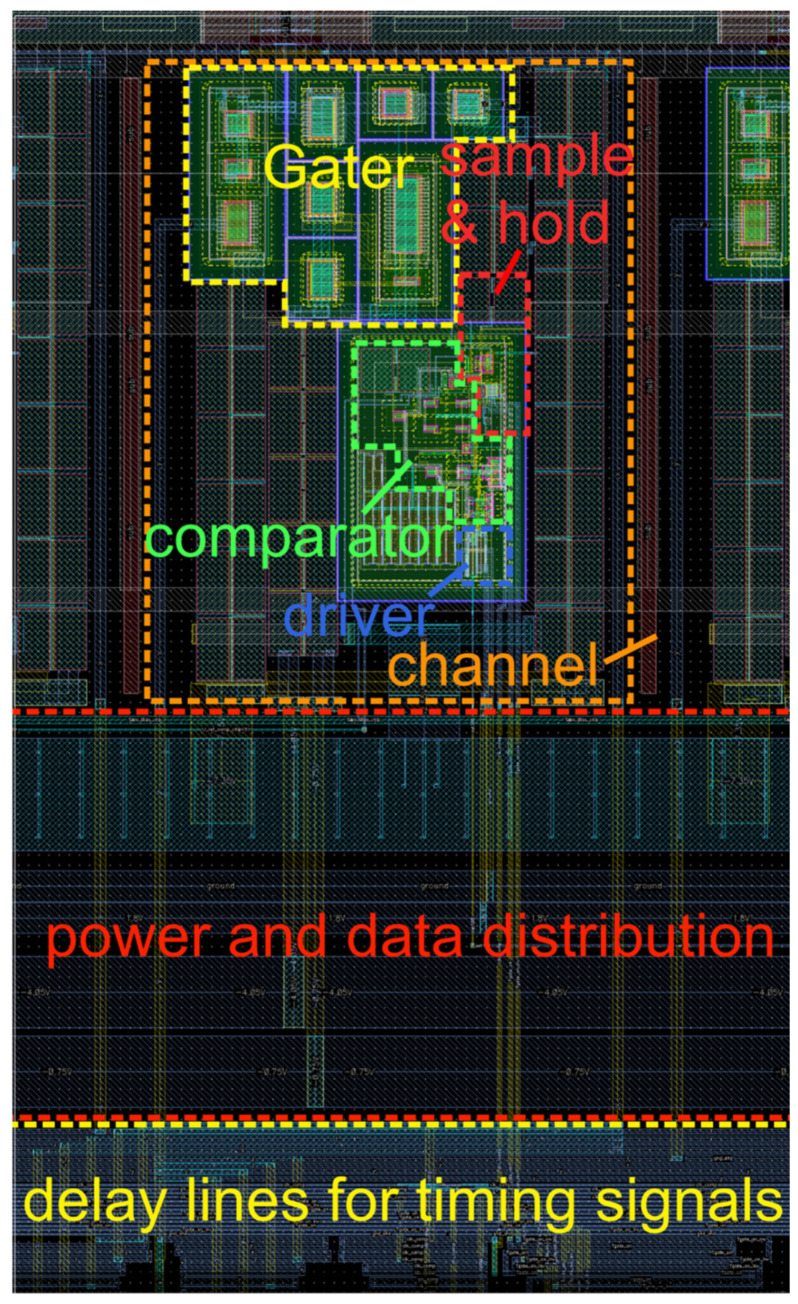
Layout of one gater channel.

The gater ASIC with the nine integrated SPADs and for coupling the photons via a fiber ribbon into the SPADs has a chip size of about 5.8 × 3 mm^2^. The layout of the complete gater chip with integrated SPADs is presented in Figure 6. The chip photo of the fabricated ASIC is depicted in Figure 7. One gating circuit with an area of 380 × 640 μm^2^ is shown in Figure 8, including parts of the power and data distribution and the delay lines. Due to the fast, simultaneous switching of all channels, high current peaks occur. The various supply voltages are therefore stabilized with large arrays of metal capacitors (MIM) and MOS capacitors on the right side shown in Figure 6.

The SPADs are placed at a spacing (pitch) of 256 µm to ensure that the intended fiber ribbon [20] fits on the chip. The fiber ribbon is the reason why the ASIC is much bigger than it would normally be because of the layout of the circuit blocks alone. The end of the fiber ribbon is mounted close to the surface of the gater ASIC for complete coupling of the light from the fiber cores into the SPADs. Therefore, bond wires and bond pads are not allowed in the area reserved for the fiber ribbon. The end of the fiber ribbon has dimensions of 10 mm × 4.25 mm. Figure 6 shows the layout of the gater ASIC with the integrated SPADs having a diameter of 50 μm. Two PIN photodiodes for easing the adjustment of the fiber ribbon are placed one at each end of the line of the nine SPADs at a distance of 127 μm. Figure 7 shows a chip photo of the gating chip glued on a PCB including the connected gold bond wires without the fiber ribbon.

### 4.4. Post-Layout Simulations

**Figure 9:**
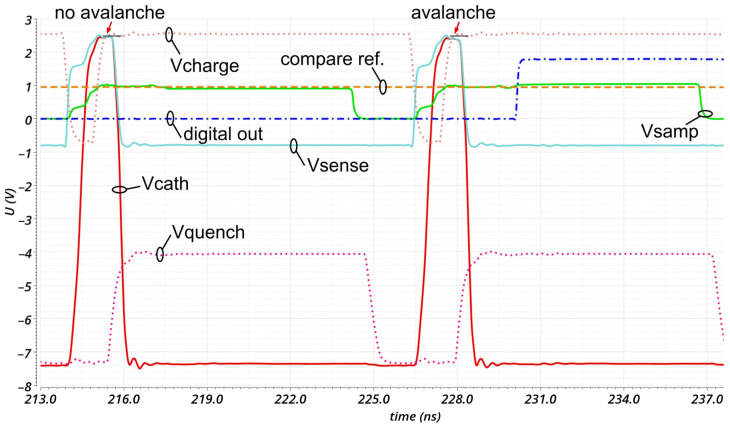
Transients at important nodes obtained by post-layout simulation.

Figure 9 shows the transient of the cathode’s voltage (red), the input of the source follower V_sense_ (turquoise), the output of the sample and hold stage V_samp_ (green), the comparator’s digital output (blue), the comparator’s reference voltage (compare ref., orange), the quenching trigger signal V_quench_ (dotted violet) and the charging trigger signal V_charge_ (dotted red) as a post-layout simulation. Due to the fully integrated design, it is not possible to measure the real transient signals.

The cathode of the SPAD is charged to 2.55 V if it is “on” and quenched to −7.55 V. The gater switch has a 10% to 90% rise time of 480 ps and a fall time of 280 ps. The swing is 9.9 V. The full width at half maximum is 1.26 ns, but can be adjusted (increased) externally with a bias voltage. Also, the charging duration can be externally adjusted with a bias voltage. The proposed channels achieve a detection sensitivity of better than 100 mV at the SPADs cathode. All channels are fully synchronized to the input clock (CLKin shown in Figure 1) and all open the gating window at the same time with a jitter of less than 50 ps. This is ensured in the timing block with a delay network and delay lines. The input clock can be up to 150 MHz. The digital output drivers can drive 50 Ohm loads. The total power dissipation of the hole ASIC is less than about 250 mW at a gating frequency of 80 MHz.

## 5. Characterization of SPAD

**Figure 10:**
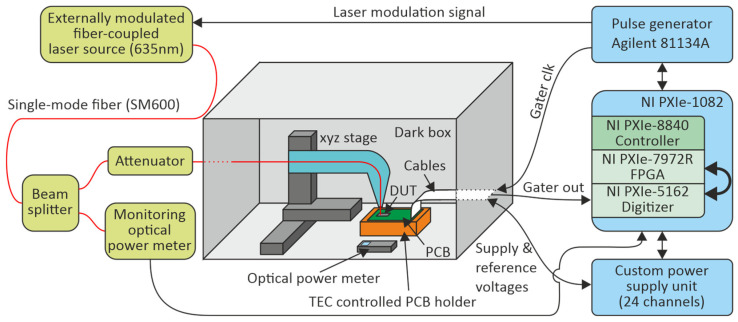
Measurement setup for characterizing the SPADs in connection with the gater chip.

The measurement setup is derived from the one presented in [21] and controlled by a PXI system from National Instruments (NI, Austin, TX, USA), comprised of the chassis NI-PXIe-1082 and the controller NI PXIe-8840 (NI), as depicted in Figure 10. Additionally, this system contains a FlexRIO FPGA card (NI PXIe-7972R), which allows streaming sampling data of a digitizer card (NI-PXIe-5162) with 625 MS/s in real time. This FPGA (field-programmable gate array) was used to extract the pulses at the output of the gater and to derive the pulse statistics for plotting the dark count rate (DCR) as well as the after-pulsing probability (APP). The APP was extracted from the inter-arrival time histograms during the dark measurements [21].

The gater chip was bonded onto a printed circuit board (PCB) using an FR4 substrate. The temperature of the gater chip was regulated to 25 °C by means of a thermoelectric cooler. Stabilizing the temperature is crucial for the characterization of SPADs, because the breakdown voltage as well as the dark count rate are particularly strongly temperature-dependent [21].

The PCB containing the gater was placed inside a dark box to block the background light. Light can be coupled into the SPADs on the gater chip using a single-mode fiber (SM600 from Thorlabs, Newton, NJ, USA). The mode field diameter of this fiber is in the range of 5µm and therefore considerably smaller than the active area of the SPAD, guaranteeing that almost all of the light is coupled into the active area. For adjusting the fiber output position on top of the SPAD, an xyz stage from Thorlabs (MTS50) was utilized.

The optical power at the output of the fiber was monitored by an optical power meter (Thorlabs PM100USB with Thorlabs S150C sensor) outside the dark box, connected to one output of a splitter, where the second output is connected to the fiber for the SPAD. The splitting ratio between these two outputs was set to 1125:1 by means of an optical attenuator in the splitting arm going to the SPAD. This large splitting ratio is required to have sufficient optical power at the optical power meter without saturation of the SPAD. The splitting ratio was calibrated before measurement using a second optical power meter inside the dark box and by placing the fiber output on top of the sensor head using the xyz stage.

A custom-built fiber-coupled laser at 635 nm was used as a light source. This laser was externally modulated by an electro-optic modulator from Jenoptic (AM635, Jena, Germany). Due to the availability of this fast light source with a high extinction ratio, the SPAD was only characterized at that wavelength. The achieved extinction ratio during the measurements was in the range of 100. The laser source was modulated by 2 ns long pulses with a repetition rate of 15 MHz. Please note, due to the limited extinction ratio, the monitored optical power does not only contain the optical power during the light pulse but also the pulse pause. This causes the optical power during the pulse to be overestimated, which results in underestimation of the extracted photon detection probability (PDP). The mean photon rate during the characterization of the PDP was set to 255,000 photons/s.

The relatively low repetition rate of 15 MHz was chosen in order to keep the after-pulsing probability low. The modulation signal for the laser, as well as the gating signal for the gaters, are generated by a pulse generator (Agilent 81134A, Santa Clara, CA, USA), which also allows accurately adjustment of the delay between these two signals, to optimize the overlap of the gating window with the arrival of the laser pulses at the SPADs.

The gating window was set to a length of ~4.5 ns, the reference voltage was optimized for each channel separately, and the substrate voltage V_sub_ was swept from −32 V to −43 V.

The dark count rate (DCR) as well as the after-pulsing probability (APP) were measured in dark conditions with a deactivated laser source. The reference voltages of the different channels were optimized for each specific channel.

**Figure 11:**
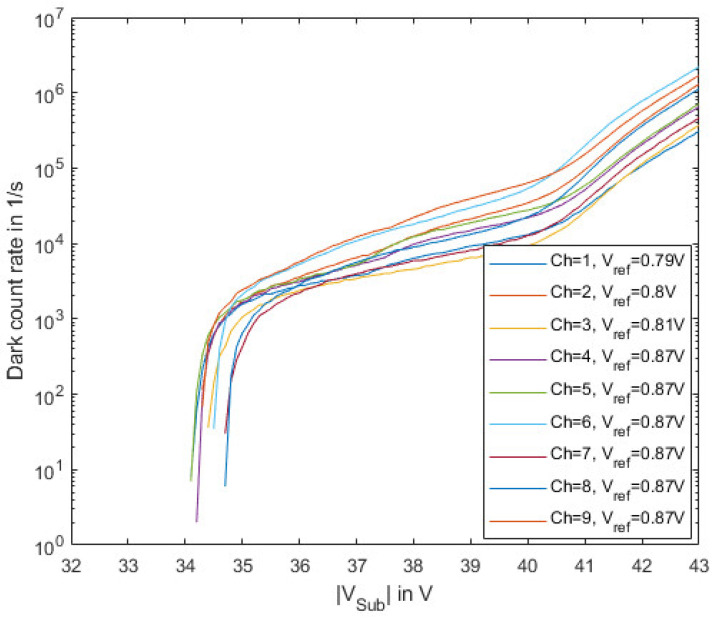
Dark count rate (DCR) depending on the substrate voltage for the nine channels of the gater chip at room temperature (25 °C).

Figure 11 shows the extracted dark count rate for the nine different channels of one gater ASIC reaching values of a few thousand to a few ten thousand counts per second in the useful operating range. It is important here to mention that the device was characterized at room temperature (25 °C). By cooling the device, the DCR can be reduced significantly, as, e.g., shown in [21]. The breakdown voltage varies by less than 1 V between the different channels. A different reference voltage for every channel can be set to achieve the best possible performance especially with respect to PDP and DCR. A larger reference voltage corresponds to a smaller detection threshold and therefore allows increasing the PDP, because also very small pulses can be detected. However, if the detection threshold is too small, noise (thermal noise and mainly power supply noise) at the input of the comparator will also generate an output signal, which would result in an increase in DCR and APP. Therefore, for each channel, the reference voltage was increased as much as possible (be aware that a larger reference voltage reduces the detection threshold) before the comparator was triggered (significantly) by noise. The first three channels (see Figure 11) need a slightly lower reference voltage V_ref_, which seems to have a systematic origin (e.g., differences in the supply of the channels due to unequal on-chip series resistances). Since the reference voltages are adjustable independently for each channel, this does not degrade the overall performance.

**Figure 12:**
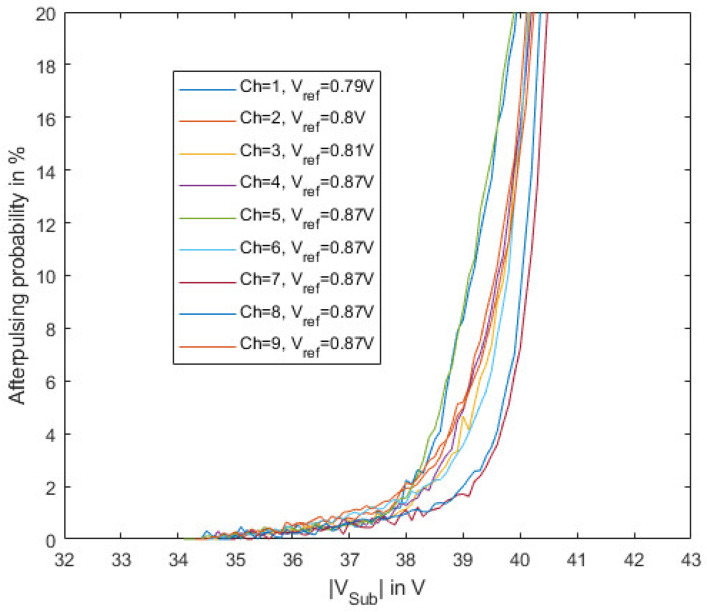
After-pulsing probability (APP) depending on the substrate voltage for the nine channels of the gater chip at room temperature (25 °C).

The after-pulsing probability was extracted by characterizing the distribution of the interarrival times between two consecutive pulses similar as in [21] and is shown in Figure 12. The resulting after-pulsing probability is quite low in the useful operating range. After-pulsing strongly depends on the dead time of the device. Therefore, further reducing the repetition rate of the gating pulses could further improve the APP, while increasing the repetition rate will lead to an increase in APP.

**Figure 13:**
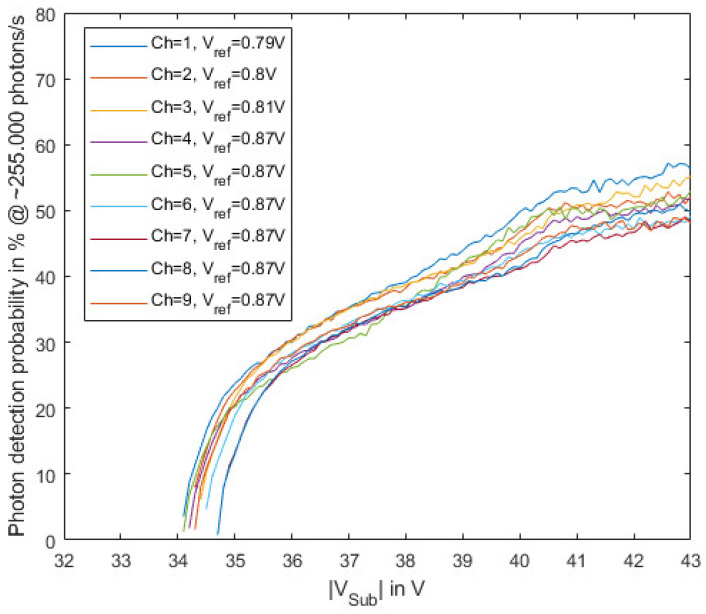
Photon detection probability (PDP) depending on the substrate voltage for the nine channels of the gater chip at room temperature (25 °C) and at a photon rate of ~255,000 photons/s with 635 nm laser source.

Figure 13 shows the extracted photon detection probability measured at a photon rate of ~255,000 photons/s. The saturation effects of the gater, as well as the influence of after-pulsing probability and dark count rate, were corrected. The limited extinction ratio of the laser was not corrected, since photons absorbed during the pulse pause might still contribute to the pulse rate if the generated charge carriers are diffusing to the absorption region while the gate window is active. Therefore, the plotted PDP will be slightly underestimated. Differences between the channels can, e.g., be explained by different transmission coefficients through the oxide stack. The SPADs in this chip do not have an anti-reflection coating, resulting in interference effects in the oxide stack, which results in a strongly wavelength-dependent PDP [22]. Due to thickness variations in the different layers in the oxide stack, this dependence can be different for the different channels. Furthermore, there is a large difference in the optical transmission between interference maxima and minima, which can occur for a certain wavelength due to oxide thickness tolerances from process run to process run or from wafer to wafer. The location of the interference maxima and minima in the spectrum can also change from run to run and wafer to wafer.

The measurement of DCR and the APP show that the channels behave similarly. The deviations depend on different defect densities within the SPADs. The differences in the PDP might indicate the tolerance of the ion implantation dose in the avalanche layer.

The achieved PDP was considerably higher than, e.g., the PDP of ~35% at 635 nm in [14], where 0.35 µm CMOS technology was used, but the maximum excess bias voltage was limited to 6.6 V.

## 6. Comparison and Conclusions

Compared to the gating circuit in the 0.35 µm CMOS exploiting simple cascoding with a maximum excess bias voltage of 6.6 V [16] and a PDP similar to that of [14] (35%), the excess bias voltage was increased to 9.9 V, raising the PDP to about 50%. In addition, the power consumption of the gater switch, comparator and output driver of [16] was about 60 mW at 100 MHz, compared to 27.8 mW per channel of the 0.18 µm CMOS gating circuit presented here. The simulated fall time for quenching from 90% to 10% of 0.28 ns corresponds to a quenching slew rate of 28.3 V/ns, which is an extraordinarily high value. The suggested gating circuit also performs better than the 0.35 µm SiGe BiCMOS gater of [23], where the comparator alone showed a switching time of 250 ps, the power consumption was 30 mW at 1 MHz count rate and the excess bias voltage was only 5 V.

The fast switching time and the short full width at half maximum of the gating pulses allow a high repetition rate of laser pulses for usage in a quantum simulator. Easy scaling of the gater chip to a higher number of channels, and therefore larger photon numbers and Q-bits, respectively, are possible.

The PDP of the integrated SPAD was much larger than the PDP of 25% of InGaAs SPADs at a 1.55 µm wavelength [24], which were exploited for quantum simulator applications (Supplementary Information in [25]). The gater chip with the integrated SPADs therefore makes much better quantum simulators possible. The mismatch between the SPADs’ breakdown voltages should be reduced and an anti-reflection coating would be highly desirable to eliminate the influence of process tolerances on the photon detection probability. Also, a reduction in the dark count rate and in the after-pulsing probability, especially above about 6 V excess bias voltage, is desirable. In a redesign, the need for different reference voltages of some channels should be eliminated to proceed with only one bond pad for V_ref_.

## Data Availability

Data are contained within the article.

## Abbreviation

SPAD	single-photon avalanche diode
ASIC	application-specific integrated circuit
PDP	photon detection probability
CMOS	complementary metal-oxide semiconductor
MOSFET	metal–oxide semiconductor field-effect transistor
APP	after-pulsing probability
HVCS	high-voltage double-cascoded switch
MIM capacitor	metal insulator metal capacitor
DCR	dark count rate
PCB	printed circuit board
PDP	photon detection probability
